# Through the scope, not the ceiling: Gender distribution among endoscopy sessions at the German Visceral Medicine Congress

**DOI:** 10.1055/a-2826-9119

**Published:** 2026-03-24

**Authors:** Lea Pueschel, Jennifer Gill, Marie-Louise Witte, Birgit Terjung, Petra Lynen Jansen, Heiner Wedemeyer, Miriam Wiestler, Henrike Lenzen

**Affiliations:** 1 9177 Department of Gastroenterology, Hepatology, Infectious Diseases and Endocrinology, Hannover Medical School, Hannover, Germany; 2 DRK Hospital Clementinenhaus, Hannover, Germany; 3 40242 Institute for Applied Data Science Hannover DATA | H, Hannover University of Applied Sciences and Arts, Hanover, Germany; 4 Department of Gastroenterology, GFO Kliniken Bonn, St. Josef Hospital, Bonn, Germany; 5 German Society of Gastroenterology, Digestive and Metabolic Diseases (DGVS), Berlin, Germany; 6 39726 Department of Gastroenterology, Hepatology, Interventional Endoscopy and Diabetology, Braunschweig Municipal Hospital, Brunswick, Germany

**Keywords:** Quality and logistical aspects, Training, Quality management

## Abstract

**Background and study aims:**

Despite awareness of the importance of diversity, equity, and inclusion (DEI) in endoscopy, structural impediments persist. Disparities include underrepresentation of women as speakers and chairs at endoscopy congresses.

**Methods:**

Data from endoscopy sessions of the annual German Visceral Medicine congresses from 2013 to 2024 were analyzed. Gender ratios of speakers, chairs, leadership, and award recipients were analyzed over 336 sessions using linear regression and the Cochran-Armitage test for trend.

**Results:**

A trend analysis over 11 years revealed significant increases in female participation as speakers (r² = 0.628, *P* = 0.004) and chairpersons (r² = 0.815, *P* < 0.001). For speakers, the increase was significant in plenary sessions (r² = 0.628, *P* = 0.004), but not in abstract sessions (r² = 0.154, *P* = 0.232) or industry symposia (r² = 0.150, *P* = 0.239). Meanwhile, female chairperson representation increased significantly across all domains: plenary sessions (r² = 0.815, *P* < 0.001), abstract sessions (r² = 0.663, *P* = 0.002), and industry symposia (r² = 0.534, *P* = 0.011). Gender parity was not reached among speakers but among chairpersons in abstract sessions and industry symposia on isolated occasions.

**Conclusions:**

In 2019, the German Society of Gastroenterology, Digestive and Metabolic Diseases Board introduced a parity resolution concerning gender balance among congress session chairs. Since then, notable progress towards achieving gender parity has been made, with a positive trend being indicative of effectiveness of current DEI measures. Nevertheless, there remains a significant lack of adequate female representation in endoscopy sessions, particularly among central congress formats and among speakers. To address this imbalance, further measures such as binding quotas, institutional support, and targeted diversity-oriented congress planning are necessary.

## Introduction

*

In Germany, overall gender parity among physicians has nearly been achieved, with 49.54% women and 50.46% men in 2023 1 . At the same time, differences in representation remain in several subspecialties and in academic leadership positions, where women still account for 13% of the highest-ranking roles in university hospitals, including leadership and professorial positions 1 . In gastroenterology, available data indicate a persistent gap: In 2024, women constituted 42% of all physicians in internal medicine but 25% of board-certified physicians in gastroenterology 2 . Data from the German Society for Gastroenterology, Digestive and Metabolic Diseases (Deutsche Gesellschaft für Gastroenterologie, Verdauungs- und Stoffwechselkrankheiten; DGVS) further contextualize this development, showing a rise in the proportion of female society members from 21% in 2018 to 28% in 2024, whereas women represented an even higher share of participants at the German Congress of Visceral Medicine in those years, with 31% in 2018 and 41% in 2024. In Germany, gastroenterology and endoscopy are typically not recorded as separate subspecialties in routine workforce statistics, which limits availability of endoscopy-specific baseline data.

Gender equity is increasingly being addressed within gastrointestinal endoscopy and its academic structures. In 2024, the European Society of Gastrointestinal Endoscopy (ESGE) published a position statement on diversity, equity and inclusion (DEI) in gastrointestinal endoscopy 3 , prompted by a survey indicating differences in participation across key society activities such as guideline development, curricula work and congress faculty roles. In that survey, several influential roles were predominantly held by men, including 77% of faculty positions, 68% of grant recipients, and 83% of guideline authors; in addition, until 2023, no women served on the ESGE Executive Committee, and women comprised 15% of the Society’s Governing Board 3 .

Beyond society structures, women continue to be underrepresented in gastroenterology journals, both in general editorial board membership and in leadership roles 4 , which has been linked to reduced publication opportunities for female authors 5 . Academic congresses represent another high-visibility arena where invitations, chairing roles, and panel participation contribute to professional recognition and career development. Prior studies assessing gender distribution among members, participants, speakers, and chairpersons at medical congresses have reported male predominance 6 7 8 , particularly in prestigious plenary sessions 9 10 and contextualize within endoscopy-related contexts 11 .

In the present analysis, gender equity in academic endoscopy was evaluated with a focus on women's roles at the German Visceral Medicine congress from 2013 to 2024. This initial measure signifies a pivotal step towards progressive institutionalization of diversity and inclusivity within academic congress planning. By establishing the foundation for prospective implementation of binding diversity quotas, in conjunction with development of comprehensive institutional support structures, the objective was to transcend mere symbolic representation, thereby striving toward attainment of systemic equity. The results of the present analysis should provide motivation for integration of diversity-oriented frameworks into the conceptualization and operationalization phases of congress organization, thereby ensuring that inclusivity becomes an integral and measurable component of academic practice rather than a marginal consideration.

## Methods

**Fig. 1:**
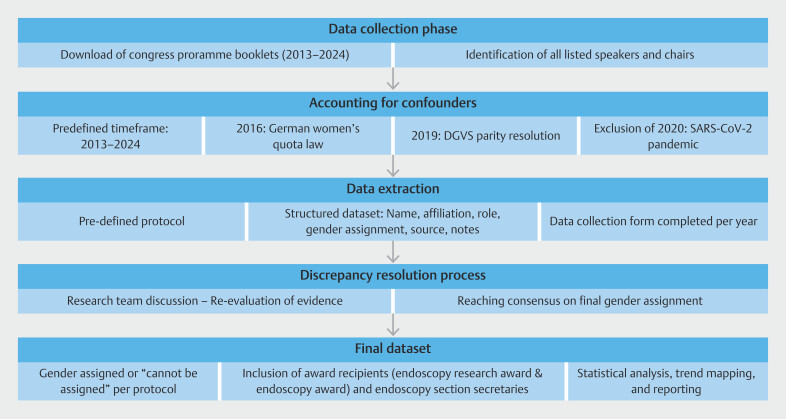
Flowchart illustrating the methodological workflow. DGVS, German Society of Gastroenterology, Digestive and Metabolic Diseases.

This investigation was based on a comprehensive dataset which was collected between December 2024 and March 2025 ( Fig. 1 ).

### Parity resolution

In May 2019, the board of the German Society of Gastroenterology, Digestive and Metabolic Diseases (DGVS) issued an official parity resolution concerning gender balance among congress session chairs. This resolution specifically called for gender parity among the chairs of the annual Visceral Medicine Congress and was formally adopted by the DGVS board, thereby extending its scope to include the Endoscopy Section.

### Selection process

Although oral and poster sessions are open to abstract submissions, speakers for the prestigious plenary sessions and industry symposia are selected by invitation only. No publicly available information exists regarding the criteria or procedures for these invitations. Similarly, the selection process for session chairs is not transparent or formally documented.

### Protocol

A predetermined internal protocol was created to establish a methodological framework for collection, analysis, and management of missing or ambiguous data, as well as establishment of cut-off dates and determination of gender. The primary objective of this analytical endeavor was to undertake a thorough examination of gender distribution in endoscopy sessions of the annual German Visceral Medicine congress from 2013 to 2024, with a particular focus on speakers and chairpersons. Overall, data from 336 endoscopy sessions were analyzed. Data synthesis was achieved through clustering, using a structured arrangement of programmed content adapted from the congress format. In the next step, data were divided into three domains: abstracts, plenary sessions, and industry symposia. Data pertaining to sessions from the German Society of Endoscopy Nurses and Associates (DEGEA) are not included in the results because DEGEA represents healthcare and nursing professionals, medical assistants, and surgical technical assistants and is not within the remit of the DGVS congress team and is also not subject to the DGVS parity resolution. The domains also were clustered for analysis in the category “Overall”. Data for each domain were separately analyzed for speakers and chairpersons.

### Missing data and bias

Individuals whose gender was recorded as “cannot be assigned” were excluded from the analysis. In total, this affected three cases (0.15%). The year 2020 was excluded because the congress did not take place in person due to the ongoing SARS-CoV-2 pandemic.

DGVS provided anonymized data on congress participants and overall membership. Because these data were not available for the entire study period, they were not incorporated into the longitudinal analyses to avoid potential bias. In addition, the available workforce and membership information is not endoscopy-specific, because gastroenterology and endoscopy are typically not recorded as separate subspecialties in routine German statistics. For contextualization, therefore, we report the first and last years with available DGVS data (2018 and 2024). In 2018, women accounted for 21% of DGVS members and 31% of congress participants. In 2024, women accounted for 28% of DGVS members and 41% of congress participants. For comparison, in 2024 women constituted 42% of all physicians in internal medicine but only 25% of board-certified physicians in gastroenterology 2 .

### Statistics

Statistical analyses were performed using GraphPad Prism, version 10.3.0 (GraphPad Software, Boston, Massachusetts, United States); additional figures were generated using Python. Temporal trends in the female proportion were assessed using linear regression. Overall trends in gender distribution over time were evaluated using the Cochran–Armitage test for trend. Gender-specific speaker distribution between plenary and abstract sessions was compared using a chi-square test with Yates’ correction. The gender-specific number of chairpersons in the year immediately before (2018) and after (2019) the DGVS statement on parity was compared using Fisher’s exact test. The term “gender distribution” refers to analyses including both men and women (e.g., between-group comparisons and overall trend analyses), whereas “female proportion” refers to analyses assessing the time trend in proportion of women among speakers and chairs (e.g., linear regression over time).

## Results

### Trend analysis of female proportion among speakers and chairpersons

**Fig. 2:**
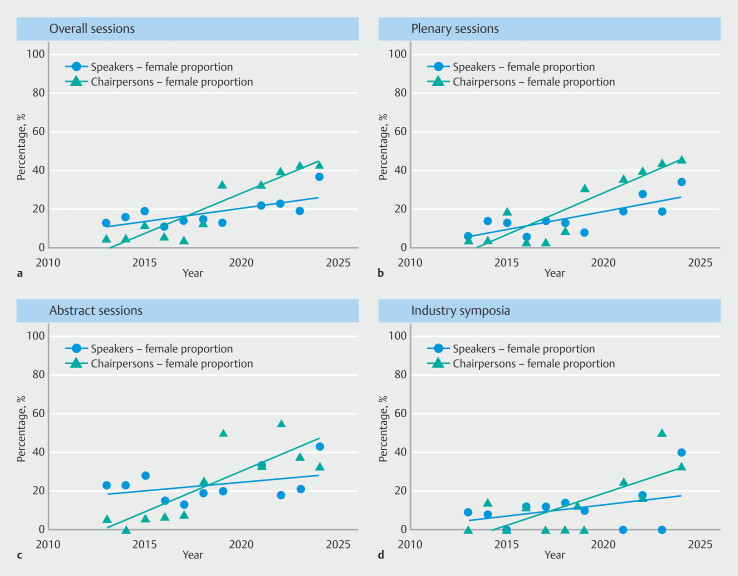
Results of linear regression analysis for trends show for **a** overall sessions a significant temporal shift for female proportions among speakers (r ^2^ = 0.497; *P* = 0.015) and chairs (r ^2^ = 0.865; *P* < 0.001); and **b** for speakers in plenary sessions (r ^2^ = 0.628; *P* = 0.004) and chairs (r ^2^ = 0.815; *P* < 0.001); but not **c** for speakers in abstract sessions (r ^2^ = 0.154; *P* = 0.232); however, for chairs (r ^2^ = 0.663; *P* = 0.002); but not **d** for speakers in industry symposia (r ^2^ = 0.150; *P* = 0.239); however, for chairs (r ^2^ = 0.534; P = 0.011).

Between 2013 and 2024, the female proportion of speakers increased significantly in the overall category (r ^2^ = 0.497; *P* =0.015), rising from 13% (19/146) in 2013 to 37% (38/103) in 2024. When stratified by session type, a significant upward trend was observed only for plenary sessions (r ^2^ = 0.628; *P* = 0.004), increasing from 6% (5/79) in 2013 to 34% (23/68) in 2024. In contrast, changes over time were not statistically significant for abstract sessions (r ^2^ = 0.154; *P* = 0.232) or industry symposia (r ^2^ = 0.150; *P* = 0.239), despite marked year-to-year variation (e.g. abstract sessions 23% [13/56] in 2013 vs 43% [12/30] in 2024; industry symposia 9% [1/11] in 2013 vs 40% [2/5] in 2024) ( Fig. 2 **a–d,** Table 1 ). Female representation among chairpersons also increased significantly in the overall category (r ^2^ = 0.865; *P* < 0.001), rising from 5% (2/44) in 2013 to 43% (15/35) in 2024. By session type, significant upward trends were observed for plenary sessions (r² = 0.815; *P* < 0.001), abstract sessions (r² = 0.663; *P* = 0.002), and industry symposia (r² = 0.534; *P* = 0.011) ( Fig. 2 **a–d,** Table 1 ).

**Table:** **Table 1** Trends for gender distribution of speakers and chairs from 2013 to 2024 in endoscopy sessions at the annual German Visceral Medicine congress and comparison of gender distribution pre- and post-implementation of the DGVS parity resolution.

	Speakers			Chairpersons		
	Men	Women		Men	Women	
Domain	n (%)	n (%)	*P* *	n (%)	n (%)	*P* *
**Overall**			**< 0.001**			**< 0.001**
2013	127 (87%)	19 (13%)		42 (95%)	2 (5%)	
2014	107 (84%)	21 (16%)		41 (95%)	2 (5%)	
2015	118 (81%)	28 (19%)		43 (88%)	6 (12%)	
2016	128 (89%)	16 (11%)		51 (94%)	3 (6%)	
2017	126 (86%)	20 (14%)		51 (96%)	2 (4%)	
2018	120 (85%)	22 (15%)		41 (87%)	6 (13%)	**^†^ 0.021 **
2019	112 (87%)	17 (13%)		39 (67%)	19 (33%)	
2021	80 (78%)	23 (22%)		26 (67%)	13 (33%)	
2022	115 (77%)	35 (23%)		28 (60%)	19 (40%)	
2023	97 (81%)	23 (19%)		20 (57%)	15 (43%)	
2024	65 (63%)	38 (37%)		20 (57%)	15 (43%)	
**Plenary**			**< 0.001**			**< 0.001**
2013	74 (94%)	5 (6%)		22 (96%)	1 (4%)	
2014	48 (86%)	8 (14%)		25 (96%)	1 (4%)	
2015	45 (87%)	7 (13%)		22 (81%)	5 (19%)	
2016	59 (94%)	4 (6%)		31 (97%)	1 (3%)	
2017	59 (86%)	10 (14%)		34 (97%)	1 (3%)	
2018	62 (87%)	9 (13%)		29 (91%)	3 (9%)	**^†^ 0.043 **
2019	59 (92%)	5 (8%)		29 (69%)	13 (31%)	
2021	34 (81%)	8 (19%)		14 (64%)	8 (36%)	
2022	53 (72%)	21 (28%)		18 (60%)	12 (40%)	
2023	61 (81%)	14 (19%)		14 (56%)	11 (44%)	
2024	45 (66%)	23 (34%)		14 (54%)	12 (46%)	
**Abstracts**			**0.342**			**< 0.001**
2013	43 (77%)	13 (23%)		15 (94%)	1 (6%)	
2014	37 (77%)	11 (23%)		10 (100%)	0	
2015	55 (72%)	21 (28%)		15 (94%)	1 (6%)	
2016	55 (85%)	10 (15%)		13 (93%)	1 (7%)	
2017	53 (87%)	8 (13%)		11 (92%)	1 (8%)	
2018	52 (81%)	12 (19%)		9 (75%)	3 (25%)	**^†^ 0.400 **
2019	44 (80%)	11 (20%)		6 (50%)	6 (50%)	
2021	30 (67%)	15 (33%)		6 (67%)	3 (33%)	
2022	53 (82%)	12 (18%)		5 (45%)	6 (55%)	
2023	34 (79%)	9 (21%)		5 (62%)	3 (38%)	
2024	17 (57%)	13 (43%)		4 (67%)	2 (33%)	
**Industry symposia**			**0.218**			**0.059**
2013	10 (91%)	1 (9%)		5 (100%)	0	
2014	22 (92%)	2 (8%)		6 (86%)	1 (14%)	
2015	18 (100%)	0		6 (100%)	0	
2016	14 (88%)	2 (12%)		7 (88%)	1 (12%)	
2017	14 (88%)	2 (12%)		6 (100%)	0	
2018	6 (86%)	1 (14%)		3 (100%)	0	**^‡^ 0.999 **
2019	9 (90%)	1 (10%)		4 (100%)	0	
2021	16 (100%)	0		6 (75%)	2 (25%)	
2022	9 (82%)	2 (18%)		5 (83%)	1 (17%)	
2023	2 (100%)	0		1 (50%)	1 (50%)	
2024	3 (60%)	2 (40%)		2 (67%)	1 (33%)	
*Cochran-Armitage test for trend. †Fisher's exact test. DGVS, German Society of Gastroenterology, Digestive and Metabolic Diseases.						

### Parity dynamics – Women as speakers and chairpersons over time

**Fig. 3:**
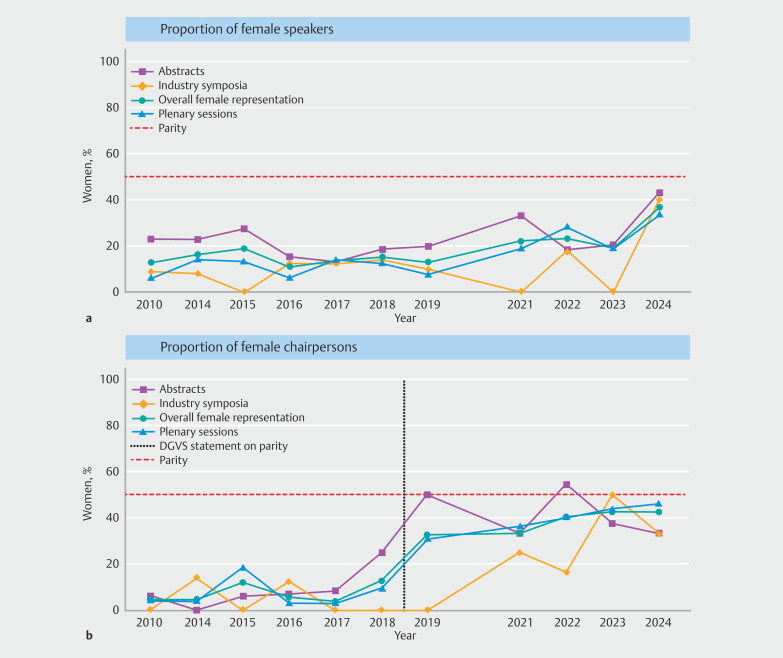
Trends in percentual representation of women among **a** speakers from 2013 to 2024, excluding 2020; and **b** among chairpersons from 2013 to 2024, excluding 2020 in Endoscopy sessions.

From 2013 to 2024, speaker representation did not reach parity in endoscopy sessions at the German Visceral Medicine congress. The highest overall female proportion was observed in 2024 at 37% (38/103) ( Fig. 3 **a,** Table 1 ). In contrast, parity among chairpersons was achieved on isolated occasions, including abstract sessions in 2019 (50%; 6/12) and industry symposia in 2023 (50%; 1/2). Notably, in 2022, women accounted for 55% of chairpersons (8/11) in abstract sessions ( Fig. 3 **b,** Table 1 ).

### Trend analysis of gender distribution among speakers

Further trend analysis of gender distribution among speakers from 2013 to 2024 demonstrated a significant change over time ( *P* < 0.001). From 2013 and 2019, men consistently accounted for at least 81% of all speakers, peaking at 89% (128/144) in 2016. In 2021, women exceeded 20% for the first time, reaching 22% (23/103). This upward shift continued, with women comprising 37% of speakers (38/103) by 2024.

By domain, plenary sessions showed a significant trend ( *P* < 0.001). Despite early male predominance (e.g. 94% men [74/79] in 2013), female representation rose to 28% (21/74) in 2022 and further increased to 34% (23/68) by 2024.

In abstract sessions, no significant trend was observed ( *P* = 0.342). Men predominated throughout the study period, peaking at 87% (53/61) in 2017, whereas the proportion of women varied substantially between years and reached its highest level in 2024 at 43% (13/30), approaching parity.

No statistically significant trend was observed for industry symposia ( *P* = 0.218), with male speakers consistently constituting the majority of speakers across most years (82%-100%), although female representation increased markedly in 2024 to 40% (2/5) ( Table 1 ).

### Trend analysis of gender distribution among chairpersons

Overall chairperson gender distribution changed significantly from 2013 to 2024 ( *P* < 0.001). Between 2013 and 2018, men accounted for at least 87% of chairpersons, peaking at 96% (51/53) in 2017. In 2019, women accounted for over 30% of chairs for the first time (33%) and reached 43% (15/35) in 2024.

For plenary sessions, a significant shift was observed over time ( *P* < 0.001), with female representation increasing to 46% (12/26) by 2024.

In abstract sessions, parity was reached for the first time in 2019 (50%; 6/12), and female representation peaked at 55% (6/11) in 2022; despite fluctuations, the overall trend remained significant ( *P* < 0.001). In industry symposia, no significant trend was found ( *P* = 0.059). Men predominated in most years (including 100% male in 2013, 2015, and 2017–2019), with parity observed only in 2023 (50% [1/2]) ( Table 1 ).

### Impact of the 2019 DGVS parity resolution

Following the DGVS board parity resolution in 2019, the proportion of female chairpersons in endoscopy session increased from 13% (6/47) in 2018 to 33% (19/58) in 2019 ( *P* = 0.021). In domain-specific analyses, a significant change was observed in plenary sessions (female chairpersons 9% [3/32] in 2018 vs 31% [13/42] in 2019; *P* = 0.043) but not in abstract sessions (25% [3/12] vs 50% [6/12]; *P* = 0.400) or industry symposia (0% [0/3] vs 0% [0/4]; *P* = 0.999) ( Table 1 ).

### Leadership and awards

**Fig. 4:**
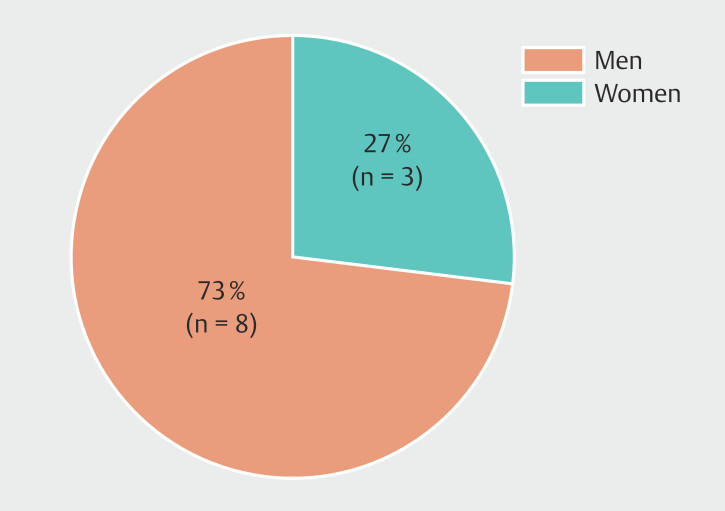
Gender distribution of the leadership position of Endoscopy Section Secretaries at the German Visceral Medicine Conference from 2013 to 2024, excluding 2020.

Between 2013 and 2024, the Endoscopy Section Secretary position was predominantly held by men (73%), with women holding the role only in 2015, 2018, and 2022 ( Fig. 4 ).

**Fig. 5:**
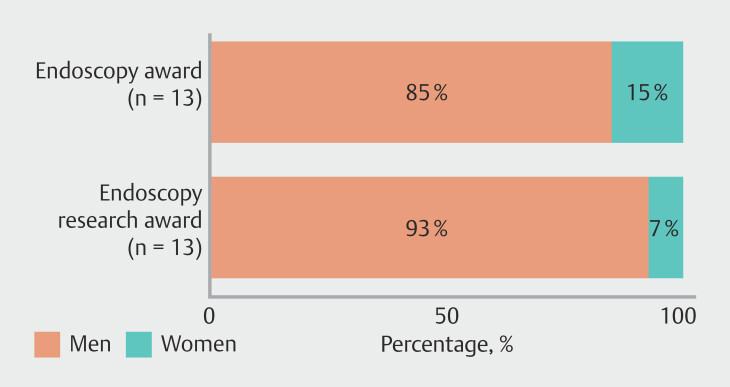
Gender distribution among award and medal recipients.

Although female representation among recipients of endoscopy-related scientific awards has increased in recent years, gender disparity persists, with male awardees remaining the majority. For the Endoscopy Research Award (n = 15), 14 of 15 recipients (93%) were men and one of 15 (7%) was a woman; for the Endoscopy Award (n = 13), 11 of 13 recipients (85%) were men and two of 13 (15%) were women. Notably, since 2017, only six of 35 applicants (17%) for the Endoscopy Research Award were women. Owing to missing data for earlier years, including for the Endoscopy Award, formal trend analyses across the full observation period were not feasible ( Fig. 5 ).

## Discussion

This analysis aimed to examine gender distribution of speakers and chairpersons in endoscopy sessions at the German Visceral Medicine congress between 2013 and 2024. Overall trend analysis of speakers indicated a shift in gender distribution over this period. Although men dominated across all session types, women were most prominently represented in abstract sessions, where female participation consistently exceeded 10% and peaked at 43% in 2024. Notwithstanding this relatively modest percentage, it was nevertheless considerably elevated in comparison with plenary sessions, where female representation ranged from only 6% to 34%. Meanwhile, in industry symposia, female representation was repeatedly at 0% (2015, 2021, 2023) and although a female speaker proportion of 40% was recorded in 2024, this increase was not attributable to a rise in the number of female speakers, but rather, to a decline in the number of male speakers.

These results are consistent with the extant literature on underrepresentation of women in prestigious speaking roles while noting that there is frequently a higher proportion of female participation in abstract presentations 12 13 14 15 16 . Indeed, a parallel evaluation for gastroenterology sessions at the German Visceral Medicine congress revealed a marked imbalance in gender distribution 10 . Considering these results, it is important to note that at the German Visceral Medicine congress, speakers for abstract sessions are selected via an application process whereas plenary and industry talks are given exclusively by invitation. This raises concerns regarding potential bias in the invitation selection process, particularly when considering extant literature demonstrating that women generally face diminished prospects of being invited to deliver high-status talks 8 . The implications and effects of this phenomenon have been extensively documented, showing that absence of women in visible and high-status speaking roles engenders diminished visibility within their fields and, thus, directly constrains career progression 17 . Moreover, it leads to a paucity of visible role models for early-career professionals, which upholds perpetuation of a cycle of underrepresentation 8 18 . It is crucial to acknowledge that pervasive gender disparity in prominent speaking roles serves to perpetuate the perception of males as the prevailing experts in the field, thereby further entrenching gender bias within the medical domain 8 19 . This phenomenon is particularly deleterious because it impedes diversity of perspectives in both research discourse and clinical guidelines. The narrow scope of scientific inquiry and innovation that ensues is likely to be detrimental, resulting in an insufficient focus on subjects pertinent to women’s health as well as underrepresented populations 20 21 22 . In the specific area of endoscopy, it has been demonstrated that congruence between endoscopist and patient gender results in enhanced health-related outcomes, with additional documented evidence indicating gender-related disparities in patient preferences, with female patients demonstrating a stronger preference for female physicians when selecting endoscopists 23 24 25 .

In the present analysis, poor visibility is highlighted by the low level of female representation among Endoscopy Award recipients. At the annual German Visceral Medicine congress, data show that female researchers submit applications for awards at a lower rate than male counterparts and are less likely to receive one. Reduced likelihood of female researchers receiving prestigious awards has been linked to visibility of their work because studies have shown that women's research is often under-cited. This under-citation is partly due to deeply ingrained gender schemas, i.e. stereotypes portraying women as less competent in scientific fields. These schemas risk creating a self-perpetuating feedback loop, systematically undervaluing women's work and reducing their chances of winning prestigious awards 26 27 28 . Conversely, reduced probability of receiving prestigious awards also acts as a hindrance to professional advancement.

Regarding gender distribution of chairpersons, the present analysis was able to demonstrate the significant impact of implementation of the DGVS board parity resolution in 2019. Since then, the proportion of female chairpersons in endoscopy sessions has overall increased to at least 30%, and achieved parity on one occasion for abstract sessions and on another occasion for industry symposia. Furthermore, in 2022, the proportion of female chairpersons in abstract sessions reached 55%. This positive development warrants emphasis because evidence suggests that organizations and professional societies that actively promote inclusion of women as organizers and leaders at conferences tend to achieve greater female representation among speakers 17 29 . Indeed, in 2022, the position of Endoscopy Section Secretary was held by a woman and incidentally, that was the first year that female representation surpassed 20% for the first time (28%) as speakers in prestigious plenary sessions. In this context, it may also be worthwhile to consider implementation of a parity resolution not only for speaker selection but also leadership roles, similar to the one that has proven effective for chairperson appointments.

Overall, it is important to recognize that gender inequity in endoscopy extends far beyond congresses. Documented evidence indicates a lack of female physicians within the domain of advanced therapeutic endoscopy 30 . Meanwhile, a comprehensive review has underscored pervasive underrepresentation of women not only in advanced endoscopy fellowship (AEF) training programs, but also among faculties for AEF, program directors, and endoscopy unit leaders 31 . These findings highlight the need for structural and systemic reforms to achieve lasting gender parity. To uphold scientific and medical rigor, congress programming must follow formal structures; thus, women require equal representation in decision-making bodies, such as scientific committees defining content and speaker selection. Achieving this demands governance, procedural, and cultural changes supported by transparent monitoring. The literature underscores strategies such as increasing women’s participation on organizing committees, appointing them as chairs or directors, and embedding gender-equity criteria in conference policies 14 32 33 . Transparent, structured speaker selection and open calls should promote diverse representation, with mixed-gender panels as the standard and all-male panels requiring justification 8 34 . Mentorship schemes preparing women for leadership roles, alongside removal of barriers such as scheduling conflicts, childcare shortages, and travel costs, enhance participation 35 36 . Conferences should also advance institutional reform through codes of conduct, visible diversity, and use of tools such as scorecards or accreditation systems to track gender equity and intersectionality 33 37 . Although it did not exist at the time of data collection, establishment and maintenance of a corresponding committee is planned for 2026 and onwards. This planned change is necessary and forward-looking, addressing both gender imbalances and the disparities shown in this analysis.

### Limitations

It is crucial to address the limitations encountered by this research. First, attribution of a name to a person cannot be used as a means to define their gender identity and, therefore, gender identity is not addressed within the scope of this analysis. Introduction of potential bias is a possibility in this context. Furthermore, there is absence of data concerning gender distribution of the invited speakers and chairpersons who declined participation.

## Conclusions

Evidence has been presented suggesting that the Endoscopy Section of the German Visceral Medicine congress has made notable progress towards achieving gender parity. This is largely attributable to the 2019 parity resolution, which has led to a steady increase in the proportion of women since it was implemented. This positive trend is indicative of the effectiveness of current DEI measures. It may be regarded as an inaugural instance of success in the endeavor to achieve inclusivity, thus providing substantiation for the notion that implementation of institutionalized changes and policies can be effective. The rise in proportion of women from 2023 to 2024 is particularly pronounced, and therefore noteworthy, with a marked peak observed in 2024. To address ongoing imbalances, it is, therefore, essential to develop and implement systematic measures that promote gender equity and diversity in the field of endoscopy. Introduction of binding requirements, such as parity quotas, is imperative, alongside provision of targeted institutional support to achieve lasting structural change.

## Footnotes

* Shared last authorship.
